# Cardiovascular and Cerebrovascular Outcomes Risk Reduction Associated With Semaglutide vs Tirzepatide: A Target Trial Emulation

**DOI:** 10.1016/j.jacadv.2026.102917

**Published:** 2026-07-22

**Authors:** Ahmed Y. Azzam, Muhammed Amir Essibayi, Pranjal Rai, Hamza A. Salim, Mustafa S. Alhasan, Mohammad Anindo, Anas Hashem, Adam A. Dmytriw, David J. Altschul, Vivek S. Yedavalli, Fabricio Feltrin, Sumit Singh, James Milburn, Dhairya A. Lakhani

**Affiliations:** a Department of Neuroradiology, WVU Rockefeller Neuroscience Institute, West Virginia University, Morgantown, West Virginia, USA; b Department of Neurological Surgery and Montefiore-Einstein Cerebrovascular Research Lab, Montefiore Medical Center, Albert Einstein College of Medicine, Bronx, New York, USA; c Department of Radiology, Mayo Clinic, Rochester, Minnesota, USA; d Department of Neuroradiology, MD Anderson Medical Center, Houston, Texas, USA; e Radiology Division, King Khaled Eye Specialist Hospital & Research Center, Riyadh, Saudi Arabia; f Consultant Teleradiologist, Teleradiology Solutions, Ardmore, Pennsylvania, USA; g Department of Hospital Medicine, Hattiesburg Clinic, Hattiesburg, Mississippi, USA; h Department of Cardiology, Houston Methodist Hospital, Houston, Texas, USA; i Neuroendovascular Program, Massachusetts General Hospital, Harvard University, Boston, Massachusetts, USA; j Nuffield Department of Surgical Sciences, Medical Sciences Division, University of Oxford, Oxford, United Kingdom; k Neurointerventional & Neuroanalytics Consortium (NAN-C), School of Medicine, Toronto Metropolitan University, Toronto, Ontario, Canada; l The Russell H. Morgan Department of Radiology and Radiological Science, Johns Hopkins University, Baltimore, Maryland, USA; m Division of Radiology – Neuroradiology, University of Texas Southwestern Medical Center, Dallas, Texas, USA; n The University of Queensland Medical School, Ochsner Clinical School, New Orleans, Louisiana, USA; o Department of Radiology, Ochsner Clinic Foundation, New Orleans, Louisiana, USA; p Department of Neuroscience, WVU Rockefeller Neuroscience Institute, West Virginia University, Morgantown, West Virginia, USA

**Keywords:** cardiovascular outcomes, cerebrovascular outcomes, semaglutide, tirzepatide, type 2 diabetes

## Abstract

**Background:**

Glucagon-like peptide-1 receptor agonists demonstrate cardiovascular benefits; however, head-to-head comparisons between semaglutide and tirzepatide remain limited.

**Objectives:**

Whether treatment effects differ by diabetes status is still limited in the current literature evidence. We compared cardiovascular outcomes between these agents stratified by type 2 diabetes status using target trial emulation approach.

**Methods:**

We conducted a target trial emulation study following TARGET framework of 217,920 adults initiating semaglutide or tirzepatide based on TriNetX Research Network Platform. Patients were stratified by diabetes status and matched 1:1 on baseline characteristics (type 2 diabetes: 48,507 pairs; nondiabetic: 60,453 pairs). Primary outcomes included atrial fibrillation, heart failure (HF), and acute myocardial infarction. Secondary outcomes included ischemic stroke/transient ischemic attack, hemorrhagic stroke, and peripheral artery disease. Follow-up extended to 3 years.

**Results:**

Tirzepatide was associated with a lower risk across all outcomes at 1 and 3 years. For HF at 1 year, risk ratios were 0.82 (95% CI: 0.78-0.86) in diabetic and 0.60 (0.55-0.65) in nondiabetic patients, with risk differences of 1.5% and 0.9%, respectively. Effect modification by diabetes status was significant for atrial fibrillation (*P* = 0.003), HF (*P* < 0.001), and acute myocardial infarction (*P* = 0.019) at 1 year. Risk reduction associations appeared to strengthen over the 3-year follow-up period.

**Conclusions:**

Tirzepatide was associated with more favorable risk reduction compared with semaglutide across multiple outcomes, with effect heterogeneity by diabetes status suggesting differential treatment associations across these populations.

Cardiovascular disease remains the leading cause of morbidity and mortality among individuals with obesity and type 2 diabetes mellitus, necessitating therapeutic strategies that address both metabolic dysfunction and cardiovascular risk. The emergence of glucagon-like peptide-1 receptor agonists (GLP-1 RAs) has further advanced the management of type 2 diabetes and obesity, offering significant glycemic control, weight reduction, and cardiovascular benefits beyond their primary metabolic effects. Multiple randomized controlled trials (RCTs) have demonstrated cardiovascular risk reduction with GLP-1 RAs compared to placebo, in which the results are demonstrating promising evidence that they could have a significant role as a cornerstone in cardiovascular risk mitigation for high-risk populations in near future.^1^, ^2^, ^3^, ^4^

Semaglutide and tirzepatide represent commonly adopted in real-world settings within GLP-1 RAs drug classes, each demonstrating significant efficacy in glycemic control and weight management. Semaglutide has shown cardiovascular benefits in landmark trials across different populations. Tirzepatide which is a dual glucose-dependent insulinotropic polypeptide (GIP) and GLP-1 RA, represents a novel pharmacological approach which warrant proper investigation beside glycemic control as in cardiovascular and cerebrovascular outcomes. Although indirect comparisons suggest tirzepatide may offer superior weight reduction and glycemic control, direct head-to-head comparisons evaluating cardiovascular outcomes between these agents remain currently limited in real-world practice based on literature evidence.^5^^,^^6^

In addition to that, the pathophysiology and cardiovascular risk profiles differ between patients with type 2 diabetes and those without diabetes who receive these medications for weight management. Patients with diabetes have greater baseline cardiovascular risk, more extensive atherosclerotic burden, and peculiar metabolic derangements compared to nondiabetic individuals with obesity. Whether the comparative cardiovascular effects of semaglutide vs tirzepatide differ according to diabetes status, which could have possible effect modification, has not been structurally evaluated in broad scale yet. Understanding such heterogeneity is important for personalized therapeutic and risk stratification decisions in our practice.^7^, ^8^, ^9^

Given the expanding indications for GLP-1 RAs beyond diabetes management and the growing utilization of these agents across different patient populations, detailed evaluation and investigation of the comparative effectiveness evidence is with significant importance to inform better state of evidence in the modern literature that could help in shaping future trials and further directions for patients. Based on these objectives, we aim to conduct a large-scale target trial emulation study to compare risk reduction endpoints for cardiovascular outcomes and cerebrovascular outcomes between semaglutide and tirzepatide individuals, stratified by type 2 diabetes status, with extended follow-up to assess both short-term and long-term treatment effects.

## Methods

### Study design and data source

We conducted a target trial emulation based study using TriNetX Research Network Platform data with data inclusion timeframe from inception up to September 23, 2025, of indexing window in the platform research network.^10^, ^11^, ^12^, ^13^, ^14^ The study was designed and reported according to 2 major frameworks and guidelines according to TARGET framework for target trial emulation^15^, ^16^, ^17^ as well as in accordance with the Strengthening the Reporting of Observational Studies in Epidemiology (STROBE) guidelines to ensure proper methodological adherence and transparent reporting.^18^^,^^19^ Target trial emulation is a causal inference framework that structurally designs observational studies to emulate hypothetical RCTs by explicitly defining trial eligibility, treatment strategies, and outcome measures, thereby minimizing biases inherent in traditional observational analyses.

TriNetX has been granted a waiver from the Western Institutional Review Board at West Virigina University. This is because, as a federated network, any data displayed on the TriNetX Platform (whether in aggregate form or as patient-level data in a generated data set) contains only deidentified data, adhering to the deidentification standard defined in section §164.514(a) of the Health Insurance Portability and Accountability Act (HIPAA) Privacy Rule. In addition to that this retrospective study is exempt from informed consent. The data reviewed are a secondary analysis of existing data, do not involve intervention or interaction with human subjects, and are deidentified per the deidentification standard defined in section §164.514(a) of the HIPAA Privacy Rule. The process by which the data is deidentified is attested to through a formal determination by a qualified expert as defined in section §164.514(b)^1^ of the HIPAA Privacy Rule.

### Study population and eligibility criteria

The study population included adult patients who initiated treatment with either semaglutide or tirzepatide between the index date and study end. Patients were stratified into 2 cohorts based on type 2 diabetes status at treatment initiation. The type 2 diabetes cohort included individuals with documented diagnosis of type 2 diabetes mellitus before or at the time of medication initiation. The nondiabetic cohort consisted of individuals without documented diabetes who initiated these medications mainly for weight management or obesity treatment. Before propensity score matching, the type 2 diabetes group included 78,000 semaglutide users and 71,000 tirzepatide users, whereas the nondiabetic group comprised 95,000 semaglutide users and 83,000 tirzepatide users. Patients were required to have sufficient baseline data for covariate assessment and were excluded if they had prior exposure to both study medications or had inadequate follow-up data for outcome ascertainment.

### Exposure definition

The primary exposure of interest was initiation of semaglutide compared with tirzepatide. Treatment initiation was defined as the first prescription fill during the study period, with the date of first prescription serving as the index date for follow-up. Patients were analyzed according to their initial treatment assignment regardless of subsequent medication changes, adherence patterns, or discontinuation, consistent with an intention-to-treat approach that reflects real-world practice settings.

### Outcome definitions

The primary cardiovascular outcomes included atrial fibrillation (AF), heart failure (HF), and acute myocardial infarction (AMI), selected based on their clinical significance and burden in this patient population. Secondary outcomes included ischemic stroke or transient ischemic attack, hemorrhagic stroke, and peripheral artery disease (PAD) or atherosclerosis. All outcomes were identified using validated diagnostic algorithms based on The International Classification of Diseases, Tenth Revision codes directly through TriNetX Research Platform. Outcome ascertainment was conducted also using TriNetX Research Network built-in tools and functions, with standardized criteria applied uniformly across both treatment groups. We assessed outcomes at 2 time horizons, 1 year and 3 years following treatment initiation, to evaluate both short-term and longer-term comparative safety. For sensitivity analyses, we evaluated outcomes using 2 definitions, a primary analysis restricted to incident cases (excluding patients with prior history of the specific outcome) and a further detailed analysis including all events regardless of prior outcome history.

### Propensity score matching and covariate balance

To minimize confounding by indication and baseline patient characteristics, we utilized propensity score matching separately within each diabetes stratum. Propensity scores representing the predicted probability of receiving semaglutide vs tirzepatide were estimated using the built-in analytical tools and statistical functions within TriNetX Platform that included demographic characteristics, anthropometric measurements, vital signs, laboratory values, comorbidities, and prior medication use. In a specific manner, covariates included age, sex, race, ethnicity, body mass index (BMI), systolic and diastolic blood pressure, heart rate, lipid profile (total cholesterol, low-density lipoprotein cholesterol, high-density lipoprotein cholesterol, and triglycerides), glycemic markers (glucose and hemoglobin A1c), and baseline cardiovascular or cerebrovascular risk factors. Patients were matched one-to-one using automated TriNetX tool for matching without replacement with a caliper width of 0.2 SDs of the logit of the propensity score. The final matched cohorts included 48,507 pairs in the type 2 diabetes stratum and 60,453 pairs in the nondiabetic stratum, resulting in a total included sample of 217,920 individuals for our analyses. Outcomes were assessed at fixed analysis windows of 1 year and 3 years following treatment initiation, with propensity score matching performed separately at each time horizon to maximize covariate balance and statistical power. The 1-year analysis included 77,178 matched patients in the type 2 diabetes stratum and 119,892 in the nondiabetic stratum, whereas the 3-year analysis included 95,614 and 115,996 matched patients, respectively. Covariate balance after matching was assessed using standardized differences, with values below 0.10 indicating adequate balance.

### Statistical analysis

Cumulative incidence of each outcome was calculated within each time horizon using Kaplan-Meier methods. Treatment effects were quantified using multiple effect measures to provide detailed assessment of comparative safety. Risk ratios (RRs) with 95% CIs were utilized for the estimations, ORs and risk differences expressed as percentage points with corresponding 95% CI. All analyses were stratified by diabetes status to evaluate possible effect modification. We attempted to test for multiplicative interaction by including an interaction term between treatment and diabetes status in regression models, with the ratio of RRs (RRR) calculated to quantify the magnitude of effect modification on the multiplicative scale. Additive interaction was assessed by calculating the interaction contrast, defined as the difference between the risk difference in diabetic patients and the risk difference in nondiabetic patients, with statistical significance evaluated using z-tests. Detailed precision metrics including 95% CIs and *P* values were reported for all effect estimates to facilitate transparent interpretation of the results and findings.

### Sensitivity and secondary analyses

Multiple sensitivity analyses were conducted to assess the significance of findings. We compared results from the incident-cases-only analysis with those from the all-events analysis to evaluate the possible impact of prevalent disease on effect estimates, calculating attenuation ratios to quantify the degree to which inclusion of prevalent cases modified the observed treatment effects. We evaluated and investigated the temporal evolution of treatment effects by plotting 1-year against 3-year RRs for each outcome stratified by diabetes status, to allow for visualization of whether treatment effects amplified, attenuated, or remained stable over extended follow-up. Extended cardiovascular outcomes including primary hypertension, ischemic heart disease, and acute coronary syndromes were also evaluated as exploratory endpoints. All statistical tests were 2-sided with statistical significance defined as *P* < 0.05. Our analyses were performed using TriNetX Platform built-in statistical tools and functions in addition to exporting the detailed comprehensive aggregated data from the platform for further needed analyses using RStudio statistical software with R (version 4.4.2), whenever indicated with consideration for the variance estimation to account for matched design.

## Results

### Study population and matching

**Figure 1:**
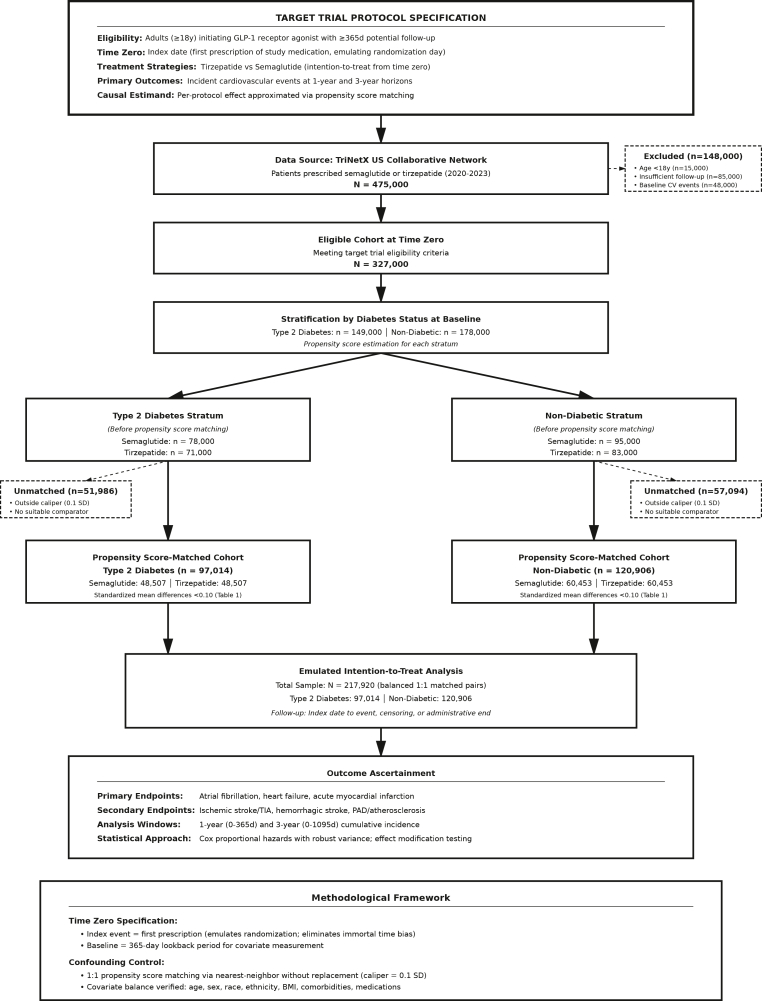
Target Trial Emulation Flow Diagram BMI = body mass index; CV = cardiovascular; GLP-1 = glucagon-like peptide-1; PAD = peripheral artery disease; TIA = transient ischemic attack.

Our cohort included 217,920 patients who initiated semaglutide or tirzepatide after propensity score matching (Figure 1). Before matching, 149,000 patients with type 2 diabetes (78,000 semaglutide users and 71,000 tirzepatide users) and 178,000 nondiabetic patients (95,000 semaglutide users and 83,000 tirzepatide users) were identified. Following one-to-one propensity score matching, the analytical cohorts included 97,014 patients with type 2 diabetes (48,507 matched pairs) and 120,906 nondiabetic patients (60,453 matched pairs).

### Baseline characteristics after propensity score matching

Baseline characteristics demonstrated significant balance between treatment groups after propensity score matching in both diabetes strata (Table 1). Among patients with type 2 diabetes, semaglutide and tirzepatide users had similar mean ages (56.0 ± 13.4 vs 56.0 ± 13.0 years, standardized difference 0.005), comparable sex distributions (54.2% vs 54.4% female, standardized difference 0.004), and similar racial composition (64.1% White in both groups, standardized difference <0.001). The mean BMI was 39.1 ± 7.1 kg/m^2^ in the semaglutide group vs 39.8 ± 7.2 kg/m^2^ in the tirzepatide group (standardized difference 0.090; *P* <0.001). Among nondiabetic patients, the cohorts were also well matched with mean ages of 47.7 ± 14.6 vs 47.8 ± 13.9 years (standardized difference 0.008), 67.7% vs 67.1% female (standardized difference 0.013), and mean BMI of 39.2 ± 7.0 vs 39.3 ± 7.0 kg/m^2^ (standardized difference 0.024, *P* <0.001).

**Table 1:** Baseline Characteristics of Propensity Score-Matched Cohorts

	Type 2 Diabetes				Nondiabetic			
	Semaglutide (n = 48,507)	Tirzepatide (n = 48,507)	Std Diff	*P* Value	Semaglutide (n = 60,453)	Tirzepatide (n = 60,453)	Std Diff	*P* Value
Demographics								
Age, years (mean ± SD)	56.0 ± 13.4	56.0 ± 13.0	0.005	0.467	47.7 ± 14.6	47.8 ± 13.9	0.008	0.162
Sex								
Female	26,279 (54.2)	26,383 (54.4)	0.004	0.503	40,937 (67.7)	40,578 (67.1)	0.013	0.028
Male	19,821 (40.9)	19,834 (40.9)	0.001	0.932	17,100 (28.3)	17,477 (28.9)	0.014	0.016
Unknown	2,407 (5.0)	2,290 (4.7)	0.011	0.080	2,416 (4.0)	2,398 (4.0)	0.002	0.791
Race								
White	31,074 (64.1)	31,079 (64.1)	<0.001	0.973	41,275 (68.3)	41,154 (68.1)	0.004	0.455
Black or African American	9,594 (19.8)	9,607 (19.8)	0.001	0.917	9,779 (16.2)	9,702 (16.0)	0.003	0.547
Asian	618 (1.3)	644 (1.3)	0.005	0.461	885 (1.5)	879 (1.5)	0.001	0.886
Other	2,739 (5.6)	2,697 (5.6)	0.004	0.558	3,087 (5.1)	3,165 (5.2)	0.006	0.311
Unknown	4,153 (8.6)	4,137 (8.5)	0.001	0.854	4,912 (8.1)	5,038 (8.3)	0.008	0.187
Ethnicity								
Hispanic or Latino	5,967 (12.3)	6,065 (12.5)	0.006	0.340	6,160 (10.2)	6,237 (10.3)	0.004	0.465
Not Hispanic or Latino	29,823 (61.5)	29,430 (60.7)	0.017	0.010	39,850 (65.9)	39,142 (64.7)	0.025	<0.001
Anthropometrics and vitals								
BMI, kg/m^2^ (mean ± SD)	39.1 ± 7.1	39.8 ± 7.2	0.090	<0.001	39.2 ± 7.0	39.3 ± 7.0	0.024	<0.001
Systolic BP, mm Hg (mean ± SD)	132.2 ± 18.2	132.1 ± 18.3	0.003	0.656	128.7 ± 16.7	128.8 ± 16.7	0.002	0.811
Diastolic BP, mm Hg (mean ± SD)	77.4 ± 11.3	77.6 ± 11.2	0.015	0.029	78.6 ± 10.6	79.1 ± 10.6	0.040	<0.001
Heart rate,/min (mean ± SD)	81.0 ± 14.2	81.0 ± 13.9	0.004	0.653	80.1 ± 13.7	80.0 ± 13.7	0.010	0.218
Lipid profile, mg/dL								
Total cholesterol (mean ± SD)	171.2 ± 45.4	170.0 ± 44.9	0.027	<0.001	185.4 ± 41.3	185.8 ± 39.9	0.010	0.152
LDL-C (mean ± SD)	94.1 ± 37.4	93.6 ± 37.1	0.015	0.039	110.1 ± 34.0	110.7 ± 33.5	0.015	0.027
HDL-C (mean ± SD)	39.5 ± 17.7	40.5 ± 17.3	0.054	<0.001	44.7 ± 19.5	45.6 ± 18.4	0.050	<0.001
Triglycerides (mean ± SD)	178.0 ± 157.2	173.9 ± 137.8	0.028	<0.001	138.7 ± 91.6	136.7 ± 86.0	0.022	0.001
Glycemic profile								
Glucose, mg/dL (mean ± SD)	156.4 ± 70.3	151.6 ± 68.4	0.070	<0.001	101.6 ± 27.9	100.9 ± 25.9	0.027	<0.001
Hemoglobin A1c, % (mean ± SD)	7.6 ± 1.9	7.5 ± 1.9	0.087	<0.001	5.7 ± 1.1	5.7 ± 1.1	0.013	0.067

BMI = body mass index; BP = blood pressure; HDL-C = high-density lipoprotein cholesterol; LDL-C = low-density lipoprotein cholesterol; Std diff = standardized difference.

### Primary cardiovascular outcomes

**Figure 2:**
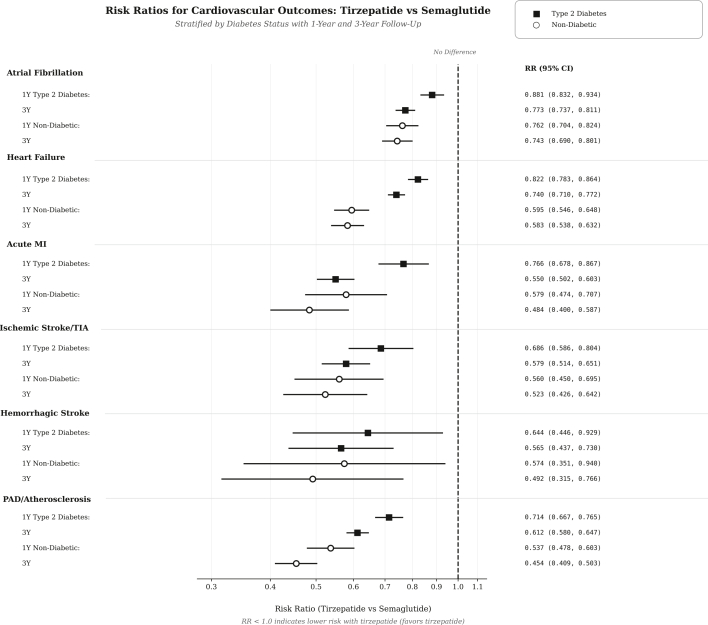
Comparative Risk Ratio for All Outcomes Forest Plot MI = myocardial infarction; RR = risk ratio; other abbreviations as in Figure 1.

Tirzepatide was associated with lower cardiovascular event rates compared with semaglutide across all primary outcomes and both follow-up periods (Table 2). For AF at 1 year, tirzepatide was associated with lower cumulative incidence of 5.4% vs 6.1% among diabetic patients (RR: 0.881; 95% CI: 0.832-0.934; risk difference 0.7%; *P* <0.001) and 1.7% vs 2.4% among nondiabetic patients (RR: 0.762; 95% CI: 0.704-0.824; risk difference 0.6%; *P* <0.001). At 3 years, diabetic patients showed 5.7% vs 7.4% (RR: 0.773; 95% CI: 0.737-0.811; risk difference 1.7%; *P* <0.001) and nondiabetic patients 2.0% vs 2.7% (RR: 0.743; 95% CI: 0.690-0.801; risk difference 0.7%; *P* <0.001) (Figure 2).

**Table 2:** Total Events for Cardiovascular Outcomes by Diabetes Status

Outcome	Time Interval	Diabetes Status	Semaglutide Risk (%)	Tirzepatide Risk (%)	Risk Ratio (95% CI)	Risk Difference, % (95% CI)	*P* Value
Atrial fibrillation	1-year	Diabetic	6.1	5.4	0.881 (0.832–0.934)	0.7 (0.4–1.1)	<0.001
		Nondiabetic	2.4	1.7	0.762 (0.704–0.824)	0.6 (0.4–0.7)	<0.001
	3-year	Diabetic	7.4	5.7	0.773 (0.737–0.811)	1.7 (1.4–2.0)	<0.001
		Nondiabetic	2.7	2.0	0.743 (0.690–0.801)	0.7 (0.5–0.9)	<0.001
Heart failure	1-year	Diabetic	8.3	6.8	0.822 (0.783–0.864)	1.5 (1.1–1.8)	<0.001
		Nondiabetic	2.3	1.4	0.595 (0.546–0.648)	0.9 (0.8–1.1)	<0.001
	3-year	Diabetic	10.1	7.5	0.740 (0.710–0.772)	2.6 (2.3–3.0)	<0.001
		Nondiabetic	2.7	1.6	0.583 (0.538–0.632)	1.1 (1.0–1.3)	<0.001
Acute myocardial infarction	1-year	Diabetic	1.5	1.1	0.766 (0.678–0.867)	0.3 (0.2–0.5)	<0.001
		Nondiabetic	0.4	0.2	0.579 (0.474–0.707)	0.2 (0.1–0.2)	<0.001
	3-year	Diabetic	2.6	1.5	0.550 (0.502–0.603)	1.2 (1.0–1.4)	<0.001
		Nondiabetic	0.6	0.3	0.484 (0.400–0.587)	0.3 (0.2–0.4)	<0.001

Risks are cumulative incidence within each time horizon from propensity score-matched cohorts. Risk ratio <1.0 indicates lower risk with tirzepatide compared with semaglutide. Risk difference calculated as semaglutide risk minus tirzepatide risk; positive values favor tirzepatide.

**Figure 3:**
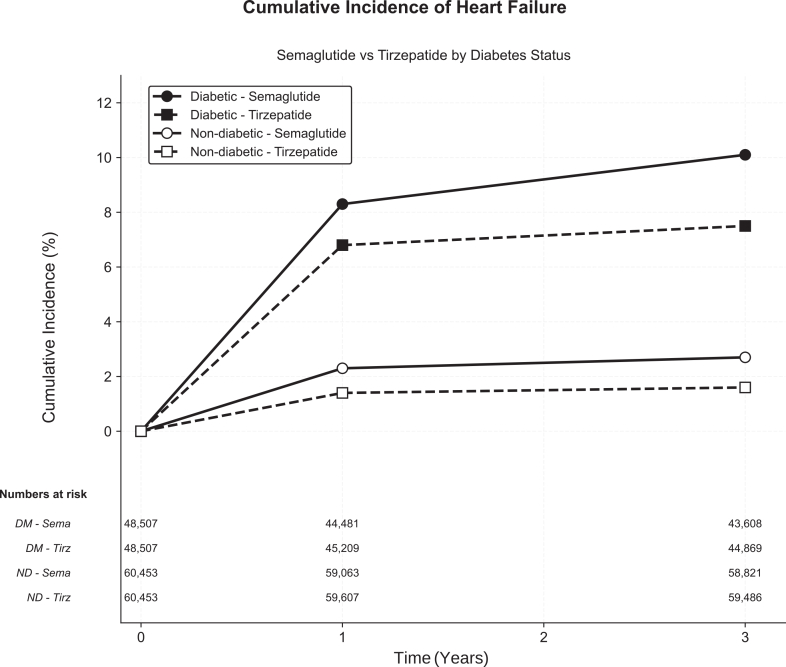
Cumulative Incidence of Heart Failure Curve

Tirzepatide was associated with significantly lower HF incidence (Figure 3). At 1 year, tirzepatide was associated with lower cumulative incidence of 6.8% vs 8.3% among diabetic patients (RR: 0.822; 95% CI: 0.783-0.864; risk difference 1.5%; *P* <0.001) and 1.4% vs 2.3% among nondiabetic patients (RR: 0.595; 95% CI: 0.546-0.648; risk difference 0.9%; *P* <0.001). By 3 years, tirzepatide was associated with incidence of 7.5% vs 10.1% among diabetic patients (RR: 0.740; 95% CI: 0.710-0.772; risk difference 2.6%; *P* <0.001) and 1.6% vs 2.7% among nondiabetic patients (RR: 0.583; 95% CI: 0.538-0.632; risk difference 1.1%; *P* <0.001).

For AMI, tirzepatide was associated with lower 1-year cumulative incidence of 1.1% vs 1.5% in diabetic patients (RR: 0.766; 95% CI: 0.678-0.867; risk difference 0.3%; *P* <0.001) and 0.2% vs 0.4% in nondiabetic patients (RR: 0.579; 95% CI: 0.474-0.707; risk difference 0.2%; *P* <0.001). At 3 years, tirzepatide was associated with the incidence of 1.5% vs 2.6% among diabetic patients (RR: 0.550; 95% CI: 0.502-0.603; risk difference 1.2%; *P* <0.001) and 0.3% vs 0.6% among nondiabetic patients (RR: 0.484; 95% CI: 0.400-0.587; risk difference 0.3%; *P* <0.001).

### Cerebrovascular and peripheral vascular outcomes

**Figure 4:**
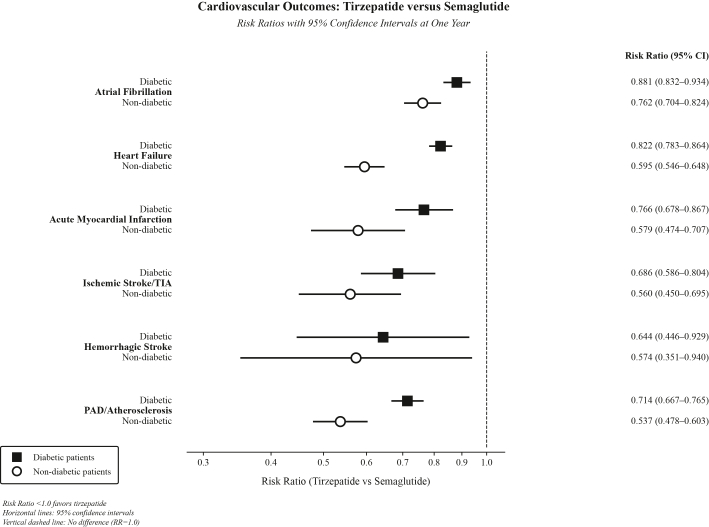
Comparative Outcomes at 1-Year Follow-up Interval Plot Abbreviations as in Figures 1 and 2.

Secondary vascular outcomes showed consistent patterns of lower event rates with tirzepatide (Table 3). For ischemic stroke or transient ischemic attack, tirzepatide was associated with lower 1-year cumulative incidence of 0.7% vs 1.0% in diabetic patients (RR: 0.686; 95% CI: 0.586-0.804; risk difference 0.3%, *P* <0.001) and 0.2% vs 0.4% in nondiabetic patients (RR: 0.560; 95% CI: 0.450-0.695; risk difference 0.2%; *P* <0.001). Hemorrhagic stroke showed lower rates at 1 year with tirzepatide: 0.1% vs 0.2% in diabetic patients (RR: 0.644; 95% CI: 0.446-0.929; *P* = 0.018) and 0.0% vs 0.1% in nondiabetic patients (RR: 0.574; 95% CI: 0.351-0.940; *P* = 0.022). PAD showed significant differences, with tirzepatide associated with 3-year incidence of 4.1% vs 6.7% in diabetic patients (RR: 0.612; 95% CI: 0.580-0.647; risk difference 2.6%; *P* <0.001) and 0.9% vs 2.0% in nondiabetic patients (RR: 0.454; 95% CI: 0.409-0.503; risk difference 1.1%; *P* <0.001) (Figure 2). A comparative summary of all cardiovascular and cerebrovascular outcomes at 1 year is presented in Figure 4.

**Table 3:** Total Events for Cerebrovascular Outcomes and Peripheral Vascular Disease by Diabetes Status

Outcome	Time Interval	Diabetes Status	Semaglutide Risk (%)	Tirzepatide Risk (%)	Risk Ratio (95% CI)	Risk Difference, % (95% CI)	*P* Value
Ischemic stroke/TIA	1-year	Diabetic	1.0	0.7	0.686 (0.586–0.804)	0.3 (0.2–0.4)	<0.001
		Nondiabetic	0.4	0.2	0.560 (0.450–0.695)	0.2 (0.1–0.3)	<0.001
	3-year	Diabetic	1.5	0.9	0.579 (0.514–0.651)	0.7 (0.5–0.8)	<0.001
		Nondiabetic	0.5	0.2	0.523 (0.426–0.642)	0.2 (0.1–0.3)	<0.001
Hemorrhagic stroke	1-year	Diabetic	0.2	0.1	0.644 (0.446–0.929)	0.1 (0.0–0.1)	0.018
		Nondiabetic	0.1	0.0	0.574 (0.351–0.940)	0.0 (0.0–0.1)	0.022
	3-year	Diabetic	0.3	0.2	0.565 (0.437–0.730)	0.1 (0.1–0.2)	<0.001
		Nondiabetic	0.1	0.1	0.492 (0.315–0.766)	0.1 (0.0–0.1)	0.001
PAD/atherosclerosis	1-year	Diabetic	4.9	3.5	0.714 (0.667–0.765)	1.4 (1.1–1.7)	<0.001
		Nondiabetic	1.4	0.7	0.537 (0.478–0.603)	0.6 (0.5–0.7)	<0.001
	3-year	Diabetic	6.7	4.1	0.612 (0.580–0.647)	2.6 (2.3–2.9)	<0.001
		Nondiabetic	2.0	0.9	0.454 (0.409–0.503)	1.1 (0.9–1.2)	<0.001

PAD = peripheral artery disease; TIA = transient ischemic attack.

Risks are cumulative incidence within each time horizon from propensity score-matched cohorts. Risk ratio <1.0 indicates lower risk with tirzepatide compared with semaglutide. Risk difference calculated as semaglutide risk minus tirzepatide risk; positive values favor tirzepatide.

### Effect modification by diabetes status

**Figure 5:**
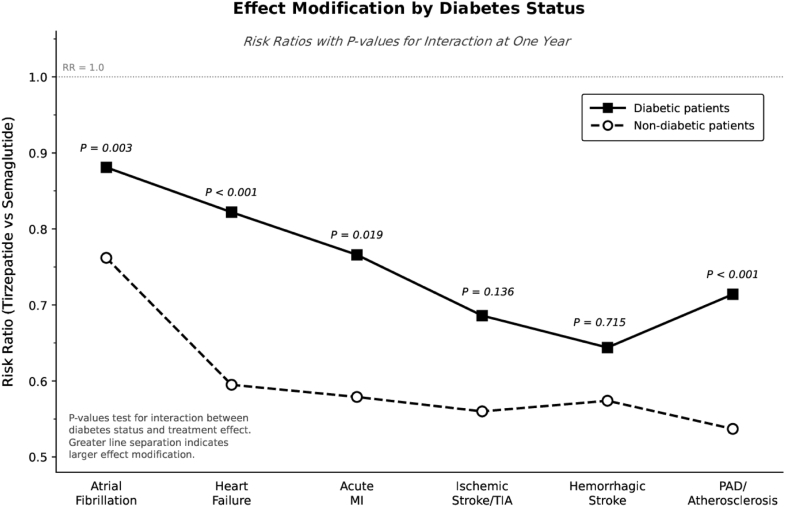
Effect Modification by Diabetes Status Plot Abbreviations as in Figures 1 and 2.

**Figure 6:**
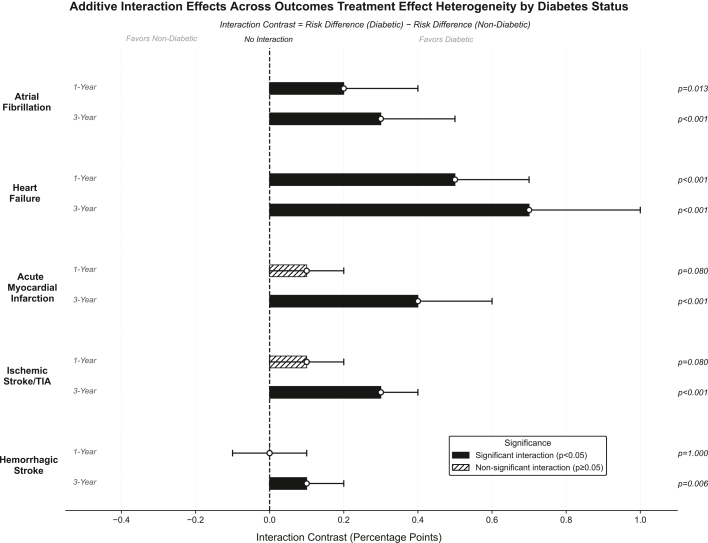
Additive Interaction Effects Across Outcomes Plot Abbreviation as in Figure 1.

A significant effect modification by diabetes status was observed at 1 year for AF (RRR: 1.157; 95% CI: 1.049-1.276; *P* = 0.003), HF (RRR: 1.383; 95% CI: 1.253-1.527; *P* <0.001), AMI (RRR: 1.325; 95% CI: 1.047-1.675; *P* = 0.019), and PAD (RRR: 1.332; 95% CI: 1.164-1.522; *P* <0.001), with nondiabetic patients showing larger relative risk reductions (Table 4). At 3 years, HF maintained significant modification (RRR: 1.269; 95% CI: 1.159-1.391; *P* <0.001) and PAD remained significant (RRR: 1.350; 95% CI: 1.202-1.517; *P* <0.001) (Figure 5). Additive interaction analysis on the risk difference scale showed consistent patterns, with diabetic patients demonstrating greater absolute treatment differences for most outcomes (Figure 6).

**Table 4:** Effect Modification by Diabetes Status - Ratio of Risk Ratios

Outcome	Time Interval	RR Diabetic (95% CI)	RR Nondiabetic (95% CI)	RRR (95% CI)	*P* Value for Interaction
Atrial fibrillation	1-year	0.881 (0.832–0.934)	0.762 (0.704–0.824)	1.157 (1.049–1.276)	0.003
	3-year	0.773 (0.737–0.811)	0.743 (0.690–0.801)	1.041 (0.951–1.136)	0.384
Heart failure	1-year	0.822 (0.783–0.864)	0.595 (0.546–0.648)	1.383 (1.253–1.527)	<0.001
	3-year	0.740 (0.710–0.772)	0.583 (0.538–0.632)	1.269 (1.159–1.391)	<0.001
Acute myocardial infarction	1-year	0.766 (0.678–0.867)	0.579 (0.474–0.707)	1.325 (1.047–1.675)	0.019
	3-year	0.550 (0.502–0.603)	0.484 (0.400–0.587)	1.136 (0.919–1.404)	0.238
Ischemic stroke/TIA	1-year	0.686 (0.586–0.804)	0.560 (0.450–0.695)	1.227 (0.938–1.605)	0.136
	3-year	0.579 (0.514, 0.651)	0.523 (0.426, 0.642)	1.107 (0.874, 1.404)	0.398
Hemorrhagic stroke	1-Year	0.644 (0.446–0.929)	0.574 (0.351–0.940)	1.121 (0.607–2.070)	0.708
	3-Year	0.565 (0.437–0.730)	0.492 (0.315–0.766)	1.149 (0.688–1.919)	0.836
PAD/atherosclerosis	1-Year	0.714 (0.667–0.765)	0.537 (0.478–0.603)	1.332 (1.164–1.522)	<0.001
	3-Year	0.612 (0.580–0.647)	0.454 (0.409–0.503)	1.350 (1.202–1.517)	<0.001

RRR = ratio of risk ratios; other abbreviations as in Table 3.

Risk ratios compare tirzepatide to semaglutide (RR < 1.0 indicates lower risk with tirzepatide). RRR represents the ratio of risk ratios (diabetic/nondiabetic). RRR >1.0 indicates larger relative treatment effect in nondiabetic patients compared with diabetic patients. *P* values test for multiplicative interaction between diabetes status and treatment.

### Extended cardiovascular outcomes and temporal evolution

**Figure 7:**
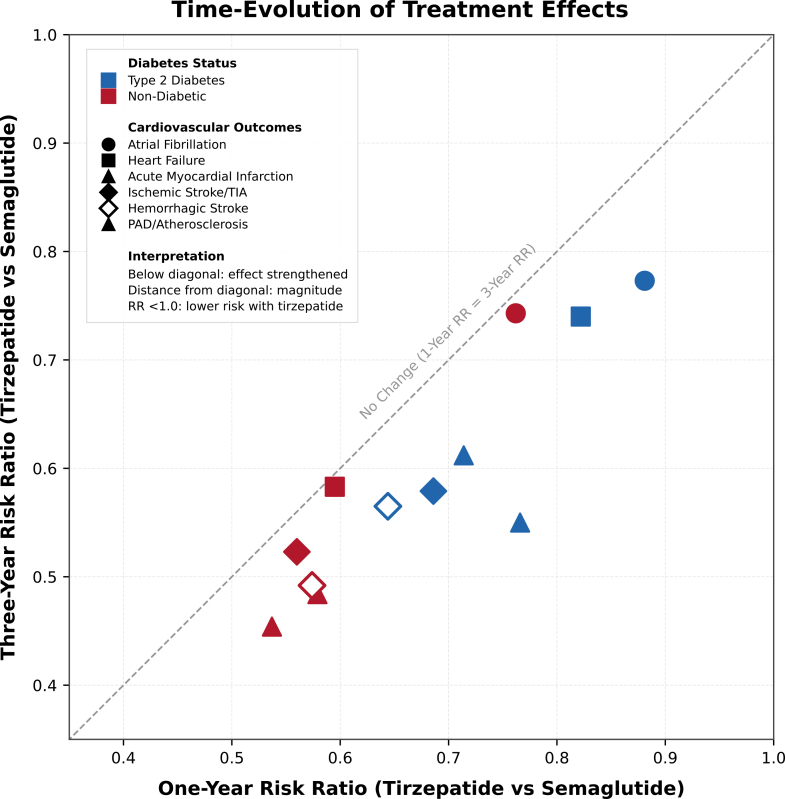
Time-Evolution of Treatment Effects Plot Abbreviations as in Figures 1 and 2.

Extended cardiovascular outcomes showed large absolute risk differences with tirzepatide (Supplemental Table 2). For primary hypertension at 3 years, tirzepatide was associated with rates of 48.2% vs 59.8% among diabetic patients (RR: 0.806; 95% CI: 0.796-0.815; risk difference 11.6%; *P* <0.001) and 22.1% vs 30.7% among nondiabetic patients (RR: 0.719; 95% CI: 0.705-0.733; risk difference 8.6%; *P* <0.001). Temporal evolution analysis suggested that tirzepatide's apparent protective association appeared to strengthen over extended follow-up, with 3-year RRs further from unity than 1-year values across nearly all outcomes (Figure 7). This pattern was particularly notable among nondiabetic patients, where the RR for AMI further decreased from 0.58 at 1 year to 0.48 at 3 years, and PAD decreased from 0.54 to 0.45, reflecting progressively greater divergence in cardiovascular risk between both agents.

## Discussion

Cardiovascular disease represents the most common cause of mortality and morbidity among individuals with obesity and type 2 diabetes, driving the imperative for therapeutic interventions that simultaneously address metabolic dysfunction and cardiovascular risk. Our large-scale target trial emulation study aimed to address the existing knowledge gap by directly comparing cardiovascular and cerebrovascular outcomes between semaglutide and tirzepatide across different endpoints, stratified by type 2 diabetes status, with extended follow-up to 3 years. Importantly, both semaglutide and tirzepatide were associated with cardiovascular and cerebrovascular risk reduction in our matched cohorts, consistent with the established class effect of GLP-1 RAs. Within this context, our findings provide real-world evidence suggesting that tirzepatide may be associated with a more favorable risk reduction profile compared with semaglutide across multiple clinically important outcomes, with significant effect modification by diabetes status (Central Illustration).^20^, ^21^, ^22^, ^23^, ^24^, ^25^

**Central Illustration:**
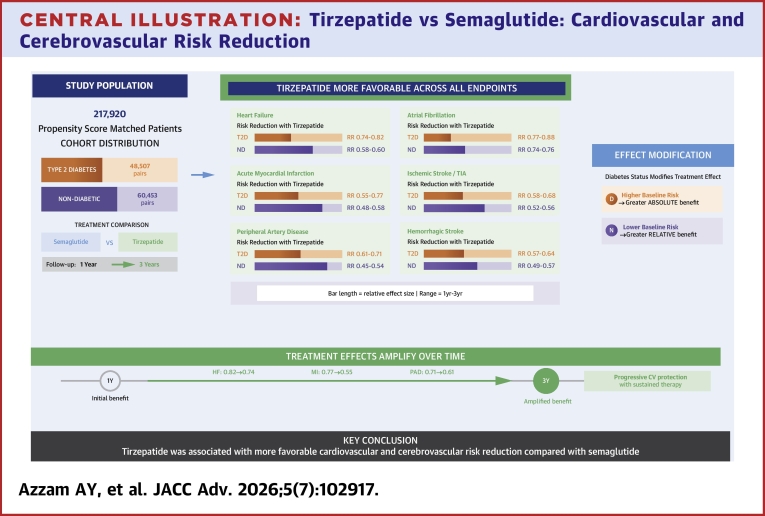
Tirzepatide vs Semaglutide: Cardiovascular and Cerebrovascular Risk Reduction Central Illustration summarizing the comparative effectiveness of tirzepatide vs semaglutide for cardiovascular and cerebrovascular risk reduction across 217,920 propensity score–matched adults (48,507 type 2 diabetes pairs; 60,453 nondiabetic pairs) followed for 1 and 3 years. Across all 6 evaluated endpoints—heart failure, atrial fibrillation, acute myocardial infarction, ischemic stroke or transient ischemic attack, peripheral artery disease, and hemorrhagic stroke—tirzepatide was associated with lower event risk compared with semaglutide, with risk ratios ranging from 0.45 to 0.88 across diabetes strata and follow-up intervals. Significant effect modification by diabetes status was observed: diabetic patients (T2D) derived greater absolute benefit owing to higher baseline cardiovascular risk, whereas nondiabetic patients (ND) experienced larger relative risk reductions. Treatment effects appeared to amplify between 1- and 3-year follow-up, suggesting progressive cardiovascular protection with sustained therapy. ND = nondiabetic; RR = risk ratio (Tirze/Sema); T2D = type 2 diabetes.

Our study of 217,920 propensity-matched patients showed consistent patterns of more favorable risk reduction estimates associated with tirzepatide compared with semaglutide across all evaluated endpoints. Among diabetic patients at 3 years, tirzepatide was associated with HF incidence of 7.5% vs 10.1%, AMI of 1.5% vs 2.6%, and AF of 5.7% vs 7.4%. Among nondiabetic patients, tirzepatide was associated with larger differences, with HF at 1.6% vs 2.7%, AMI at 0.3% vs 0.6%, and AF at 2.0% vs 2.7% at 3 years. Secondary cerebrovascular and peripheral vascular outcomes also showed favorable patterns, with tirzepatide associated with 3-year PAD incidence of 4.1% vs 6.7% in diabetic patients and 0.9% vs 2.0% in nondiabetic patients.

The significant effect modification by diabetes status emerged as a notable finding, suggesting differential treatment associations across patient populations. On the multiplicative scale, nondiabetic patients showed larger relative risk reductions with tirzepatide compared with diabetic patients. On the additive scale, diabetic patients often had greater absolute risk reductions, especially for HF where the interaction contrast reached 0.70 percentage points at 3 years. This divergence between multiplicative and additive interaction patterns reflects the higher baseline cardiovascular risk in diabetic patients, translating the observed associations into larger absolute event reductions despite smaller relative RRs.

The effect modification findings we observed suggest important insights for personalized strategies. Although nondiabetic patients experienced larger relative risk reductions, diabetic patients often derived greater absolute benefit due to higher baseline risk. This phenomenon, known as the risk paradox, has important implications, as in high-risk diabetic populations, tirzepatide's observed associations translate into larger absolute risk differences, despite smaller relative risk reductions.^6^^,^^26^^,^^27^

Our findings regarding HF deserve important focus given the emerging recognition of GLP-1 RAs' role and effects on cardiac structure and function in recent literature findings. The HF risk reduction observed with tirzepatide, ranging from 1.5% absolute reduction at 1 year in diabetic patients to 2.6% at 3 years, suggests promising cardioprotective mechanisms beyond metabolic improvements. Several biological mechanisms may underlie these observations. Tirzepatide's dual agonism of GLP-1 and GIP receptors may have additive or synergistic cardiovascular benefits through better natriuresis, improved endothelial function, reduced inflammation, and direct myocardial effects. The greater weight reduction typically achieved with tirzepatide may reduce cardiac afterload, improve diastolic function, and decrease neurohormonal activation.^28^, ^29^, ^30^, ^31^, ^32^, ^33^

In comparison with current literature evidence, our study provides direct head-to-head comparative real-world evidence that has been largely absent. The SUSTAIN-6 trial^34^ demonstrated semaglutide cardiovascular benefits compared to placebo; however, no adequately powered RCT has directly compared semaglutide and tirzepatide for cardiovascular outcomes at our estimated endpoints with extended follow-up duration yet. SURPASS program trials evaluated tirzepatide against various comparators, but these trials were designed for glycemic and weight endpoints rather than cardiovascular outcomes, with cardiovascular events captured as safety endpoints rather than primary efficacy measures. Our real-world evidence therefore fills an important present gap by providing direct comparative endpoints across different outcomes, extended follow-up periods, and both diabetic and nondiabetic populations.^35^, ^36^, ^37^, ^38^, ^39^

The temporal evolution findings we observed, with treatment associations appearing to strengthen from 1 to 3 years, go in the same direction with accumulating evidence that cardiovascular benefits of metabolic therapies may accrue over time as atherosclerotic progression is reduced, cardiac remodeling is reversed, and cumulative metabolic improvements translate into reduced cardiovascular events. This temporal pattern has important implications for treatment persistence and long-term adherence, suggesting that sustained therapy over multiple years may be necessary to realize maximal cardiovascular benefit.^40^, ^41^, ^42^, ^43^, ^44^, ^45^, ^46^, ^47^, ^48^

### Study Limitations

Despite our study proposed structured strengths, several important limitations warrant consideration. The retrospective observational study design, despite target trial emulation following TARGET framework, and control by propensity score matching, remains susceptible to residual confounding by unmeasured variables. Selection bias may impact treatment assignment, as physicians and patients' decisions may be affected by factors not fully captured. Medication adherence and persistence patterns were not captured, as we analyzed patients according to initial treatment assignment regardless of subsequent medication changes. The limited follow-up duration of 3 years remains insufficient to capture very long-term cardiovascular outcomes. In addition, the TriNetX platform employs fixed analysis time windows rather than continuous time-to-event tracking, precluding traditional median follow-up reporting; outcomes were assessed within prespecified 1-year and 3-year windows with propensity score matching performed separately at each horizon. Geographic and demographic limitations may affect generalizability, as our cohort was drawn from TriNetX research network primarily based in the United States. Outcome ascertainment through diagnostic codes, although validated and widely used, may be subject to misclassification. The observational design nor target trial emulation framework cannot formulate definitive causality, as RCTs remain the gold standard for causal inference.

Our findings highlight several important directions for future studies. Large-scale RCTs directly comparing cardiovascular outcomes between semaglutide and tirzepatide are needed to confirm our observational findings with higher-level evidence. Such trials should be adequately powered for major cardiovascular endpoints, include different patient populations, and extend follow-up to 5 years or longer. Mechanistic studies are needed to elucidate biological pathways underlying tirzepatide's apparent more favorable risk reduction profile, including effects on cardiac structure and function, vascular inflammation and endothelial function, and atherosclerotic plaque characteristics. Longer-term observational studies with follow-up extending to 5, 10, or more years would help clarify whether temporal amplification of treatment effects continues beyond our 3-year observation window. Cost-effectiveness evaluations are needed to inform health care system decisions, and comparative effectiveness studies in specific high-risk subgroups would provide valuable insights for targeted therapeutic strategies.

## Conclusions

This large-scale target trial emulation study of 217,920 patients suggests that tirzepatide may be associated with more favorable cardiovascular and cerebrovascular risk reduction compared with semaglutide across multiple endpoints, including HF, AF, AMI, stroke, and PAD. The potentially protective associations were observed across both diabetic and nondiabetic populations; however, significant effect modification by diabetes status revealed differential findings and observations of benefit, nondiabetic patients had larger relative risk reductions whereas diabetic patients had greater absolute risk reductions due to higher baseline cardiovascular risk. The treatment associations appeared to strengthen over extended follow-up from 1 to 3 years, suggesting a pattern of increasing separation with the sustained tirzepatide use.

These real-world findings have important implications for the growing literature evidence in both diabetes management and obesity. When cardiovascular risk reduction is a therapeutic priority, tirzepatide may represent a more favorable option among GLP-1 RAs based on our comparative effectiveness findings. The consistency of observed associations across different endpoints and outcomes and temporal amplification patterns collectively suggest that tirzepatide may offer more favorable risk reduction compared with semaglutide for either primary or secondary cardiovascular and cerebrovascular endpoints. Although RCTs are needed to provide definitive causal confirmation of these observational findings, our evidence provides preliminary comparative effectiveness data that may help inform therapeutic strategies for patients who are prescribed GLP-1 RAs for metabolic management and cardiovascular risk reduction.

Perspectives **COMPETENCY IN MEDICAL KNOWLEDGE:** This target trial emulation study of 217,920 propensity-matched patients provides comparative effectiveness evidence suggesting that tirzepatide may be associated with more favorable cardiovascular and cerebrovascular risk reduction compared with semaglutide across multiple endpoints, including HF, AF, AMI, ischemic stroke, and PAD. Physicians should recognize that significant effect modification by diabetes status exists, whereby nondiabetic patients experience larger relative risk reductions whereas diabetic patients derive greater absolute benefit attributable to higher baseline cardiovascular risk. Understanding the dual GIP/GLP-1 receptor agonism mechanism of tirzepatide and its potential additive cardioprotective effects beyond single-receptor agonists represents essential foundational knowledge for evidence-based prescribing.

**COMPETENCY IN PATIENT CARE AND PROCEDURAL SKILLS:** When selecting incretin-based therapies for patients with obesity or type 2 diabetes, physicians should integrate comprehensive cardiovascular risk stratification into prescribing decisions. The observed absolute risk reductions, including a 2.6 percentage-point reduction in HF incidence over 3 years among diabetic patients, may inform individualized therapeutic strategies when choosing between available agents.

**COMPETENCY IN INTERPERSONAL AND COMMUNICATION SKILLS:** Physicians should effectively communicate the differential cardiovascular benefits observed between tirzepatide and semaglutide to patients in an understandable manner, contextualizing absolute vs relative risk reductions based on individual patient risk profiles and diabetes status. Transparent discussion of the observational nature of these findings, pending confirmatory RCT data, enables patients to participate meaningfully in therapeutic decisions.

**TRANSLATIONAL OUTLOOK:** Adequately powered randomized controlled trials with prespecified cardiovascular and cerebrovascular endpoints and extended follow-up are needed to confirm whether tirzepatide confers greater cardiovascular protection than semaglutide and whether this effect differs by type 2 diabetes status. Mechanistic studies clarifying the cardioprotective pathways of dual GIP/GLP-1 receptor agonism would further inform individualized, evidence-based prescribing.

## Funding support and author disclosures

Research reported in this publication was supported by the 10.13039/100000057National Institute of General Medical Sciences of the National Institutes of Health under Award Number U54 GM104942. Dr Yedavalli is a consultant for iSchemaView, RapidAI. Dr Altschul reports securities holdings in Von Vascular, Inc, and compensation from Johnson and Johnson International, Stryker Corporation, Medtronic USA, Inc, and MicroVention, Inc, for consultant services. Dr Lakhani is consultant for iSchemaView and RapidAI; scientific advisory board member for Upstream Vision; deputy editor for Radiology: Images in Radiology; and associate editor for Frontiers in Radiology. All other authors have reported that they have no relationships relevant to the contents of this paper to disclose.
