# Supplementary material for: Cardiovascular and Cerebrovascular Outcomes Risk Reduction Associated With Semaglutide vs Tirzepatide: A Target Trial Emulation
Source: JACC Adv. 2026 Jul 22;5(7):102917. doi: 10.1016/j.jacadv.2026.102917 (PMC13400122; doi:10.1016/j.jacadv.2026.102917)
Supplement: Supplemental Tables 1-5 [file mmc1.pdf]

Supplementary Table 1: Sensitivity Analysis - Combined vs Primary-Only Outcomes.

Outcome	Time Interval	Diabetes Status	Primary + Secondary			Primary Only			Attenuation Ratio
			RR (95% CI)	RD (%)	P-value	RR (95% CI)	RD (%)	P-value	
**Atrial Fibrillation**	One-Year	Diabetic	0.881 (0.832, 0.934)	0.7	<0.001	0.696 (0.585, 0.829)	0.3	<0.001	0.43
		Non-diabetic	0.762 (0.704, 0.824)	0.6	<0.001	0.743 (0.661, 0.836)	0.4	<0.001	0.67
	Three-Year	Diabetic	0.773 (0.737, 0.811)	1.7	<0.001	0.554 (0.492, 0.624)	0.9	<0.001	0.53
		Non-diabetic	0.743 (0.690, 0.801)	0.7	<0.001	0.526 (0.461, 0.601)	0.3	<0.001	0.43
**Heart Failure**	One-Year	Diabetic	0.822 (0.783, 0.864)	1.5	<0.001	0.629 (0.547, 0.723)	0.7	<0.001	0.47
		Non-diabetic	0.595 (0.546, 0.648)	0.9	<0.001	0.733 (0.597, 0.900)	0.3	0.003	0.33
	Three-Year	Diabetic	0.740 (0.710, 0.772)	2.6	<0.001	0.513 (0.465, 0.565)	1.7	<0.001	0.65
		Non-diabetic	0.583 (0.538, 0.632)	1.1	<0.001	0.484 (0.417, 0.561)	0.4	<0.001	0.36
**Acute Myocardial Infarction**	One-Year	Diabetic	0.766 (0.678, 0.867)	0.3	<0.001	0.734 (0.593, 0.908)	0.2	0.004	0.67
		Non-diabetic	0.579 (0.474, 0.707)	0.2	<0.001	0.747 (0.593, 0.940)	0.2	0.013	1.00
	Three-Year	Diabetic	0.550 (0.502, 0.603)	1.2	<0.001	0.501 (0.428, 0.586)	0.6	<0.001	0.50
		Non-diabetic	0.484 (0.400, 0.587)	0.3	<0.001	0.445 (0.345, 0.575)	0.2	<0.001	0.67
**Ischemic Stroke/TIA**	One-Year	Diabetic	0.686 (0.586, 0.804)	0.3	<0.001	0.677 (0.522, 0.879)	0.2	0.003	0.67
		Non-diabetic	0.560 (0.450, 0.695)	0.2	<0.001	0.652 (0.477, 0.894)	0.1	0.008	0.50
	Three-Year	Diabetic	0.579 (0.514, 0.651)	0.7	<0.001	0.537 (0.454, 0.636)	0.4	<0.001	0.57
		Non-diabetic	0.523 (0.426, 0.642)	0.2	<0.001	0.461 (0.350, 0.607)	0.1	<0.001	0.50
**Hemorrhagic Stroke**	One-Year	Diabetic	0.644 (0.446, 0.929)	0.1	0.018	0.625 (0.328, 1.190)	0.0	0.149	0.00
		Non-diabetic	0.574 (0.351, 0.940)	0.0	0.022	0.546 (0.342, 0.871)	0.1	0.010	--
	Three-Year	Diabetic	0.565 (0.437, 0.730)	0.1	<0.001	0.440 (0.298, 0.650)	0.0	<0.001	0.00
		Non-diabetic	0.492 (0.315, 0.766)	0.1	0.001	0.262 (0.135, 0.509)	0.0	<0.001	0.00

Notes: *Primary + Secondary includes all patients regardless of prior outcome history; Primary only includes only incident cases. RR <1.0 indicates lower risk with tirzepatide compared with semaglutide. RD calculated as semaglutide risk minus tirzepatide risk in percentage points. Attenuation ratio calculated as RD_Primary/RD_Both when RD_Both >0.* ***Abbreviations:*** *RR, risk ratio; CI, confidence interval; RD, risk difference; TIA, transient ischemic attack.*

Supplementary Table 2: Extended Cardiovascular Outcomes For Total Events.

**Outcome**	**Time Horizon**	**Diabetes Status**	**Semaglutide Risk (%)**	**Tirzepatide Risk (%)**	**Risk Ratio (95% CI)**	**Risk Difference, % (95% CI)**	**P-value**
**Primary Hypertension**	One-Year	Diabetic	54.6	45.9	0.841 (0.827, 0.855)	8.7 (8.0, 9.4)	<0.001
		Non-diabetic	20.1	14.1	0.705 (0.690, 0.720)	5.9 (5.3, 6.3)	<0.001
	Three-Year	Diabetic	59.8	48.2	0.806 (0.796, 0.815)	11.6 (11.0, 12.3)	<0.001
		Non-diabetic	30.7	22.1	0.719 (0.705, 0.733)	8.6 (8.1, 9.1)	<0.001
**Ischemic Heart Disease**	One-Year	Diabetic	12.6	10.2	0.807 (0.775, 0.839)	2.4 (2.0, 2.9)	<0.001
		Non-diabetic	4.2	2.6	0.625 (0.587, 0.666)	1.6 (1.4, 1.9)	<0.001
	Three-Year	Diabetic	16.3	11.2	0.688 (0.667, 0.711)	5.1 (4.6, 5.5)	<0.001
		Non-diabetic	5.4	2.9	0.536 (0.506, 0.567)	2.5 (2.3, 2.7)	<0.001
**Acute Coronary Syndromes**	One-Year	Diabetic	0.34	0.16	0.462 (0.341, 0.626)	0.18 (0.10, 0.30)	<0.001
		Non-diabetic	0.10	0.04	0.439 (0.278, 0.691)	0.05 (0.00, 0.10)	<0.001
	Three-Year	Diabetic	0.59	0.24	0.403 (0.325, 0.500)	0.35 (0.30, 0.40)	<0.001
		Non-diabetic	0.14	0.05	0.366 (0.241, 0.556)	0.09 (0.10, 0.10)	<0.001

Notes: *Risks are cumulative incidence within each time horizon from propensity score-matched cohorts. Risk ratio <1.0 indicates lower risk with tirzepatide compared with semaglutide. Risk difference calculated as semaglutide risk minus tirzepatide risk; positive values favor tirzepatide. Three-year data not available for these extended outcomes.* ***Abbreviations:*** *CI, confidence interval.*

Supplementary Table 3: Additive Interaction Analysis.

Outcome	Time Horizon	RD Diabetic, % (95% CI)	RD Non-diabetic, % (95% CI)	Interaction Contrast, % (95% CI)	Z-statistic	P-value
**Atrial Fibrillation**	One-Year	0.30 (0.20, 0.50)	0.10 (0.10, 0.20)	0.20 (0.00, 0.40)	2.48	0.013
	Three-Year	0.90 (0.80, 1.10)	0.60 (0.50, 0.60)	0.30 (0.10, 0.50)	3.72	<0.001
**Heart Failure**	One-Year	0.70 (0.50, 0.90)	0.20 (0.10, 0.20)	0.50 (0.30, 0.70)	4.75	<0.001
	Three-Year	1.70 (1.50, 1.90)	1.00 (0.90, 1.20)	0.70 (0.50, 1.00)	5.49	<0.001
**Acute Myocardial Infarction**	One-Year	0.20 (0.10, 0.30)	0.10 (0.00, 0.10)	0.10 (-0.00, 0.20)	1.75	0.080
	Three-Year	0.60 (0.50, 0.80)	0.20 (0.10, 0.20)	0.40 (0.20, 0.60)	4.96	<0.001
**Ischemic Stroke/TIA**	One-Year	0.20 (0.10, 0.30)	0.10 (0.00, 0.10)	0.10 (-0.00, 0.20)	1.75	0.080
	Three-Year	0.40 (0.30, 0.50)	0.10 (0.10, 0.20)	0.30 (0.20, 0.40)	5.26	<0.001
**Hemorrhagic Stroke**	One-Year	0.00 (0.00, 0.10)	0.00 (0.00, 0.10)	0.00 (-0.10, 0.10)	0.00	1.000
	Three-Year	0.10 (0.00, 0.10)	0.00 (0.00, 0.10)	0.10 (0.00, 0.20)	2.77	0.006

Notes: *Interaction contrast (IC) = RD_Diabetic - RD_Non-diabetic, representing the difference in absolute treatment benefit between diabetes groups. Positive IC indicates greater benefit in diabetic patients. RD calculated as semaglutide risk minus tirzepatide risk in percentage points. P-values test for additive interaction on the risk difference scale.* ***Abbreviations:*** *RD, risk difference; CI, confidence interval; TIA, transient ischemic attack.*

Supplementary Table 4: Incident vs All-Events Sensitivity Analysis.

Outcome	Time Interval	Diabetes Status	Primary-only RD, %	Both Events RD, %	Delta RD, %	Attenuation Ratio
**Atrial Fibrillation**	One-Year	Diabetic	0.30	0.70	0.40	0.43
		Non-diabetic	0.10	0.60	0.50	0.17
	Three-Year	Diabetic	0.90	1.70	0.80	0.53
		Non-diabetic	--	0.70	--	--
**Heart Failure**	One-Year	Diabetic	0.70	1.50	0.80	0.47
		Non-diabetic	0.20	0.90	0.70	0.22
	Three-Year	Diabetic	1.70	2.60	0.90	0.65
		Non-diabetic	1.00	1.10	0.10	0.91
**Acute Myocardial Infarction**	One-Year	Diabetic	0.20	0.30	0.10	0.67
		Non-diabetic	0.10	0.20	0.10	0.50
	Three-Year	Diabetic	0.60	1.20	0.60	0.50
		Non-diabetic	0.20	0.30	0.10	0.67
**Ischemic Stroke/TIA**	One-Year	Diabetic	0.20	0.30	0.10	0.67
		Non-diabetic	0.10	0.20	0.10	0.50
	Three-Year	Diabetic	0.40	0.70	0.30	0.57
		Non-diabetic	0.10	0.20	0.10	0.50
**Hemorrhagic Stroke**	One-Year	Diabetic	0.00	0.10	0.10	0.00
		Non-diabetic	0.00	0.00	0.00	--
	Three-Year	Diabetic	0.10	0.10	0.00	1.00
		Non-diabetic	0.00	0.10	0.10	0.00

Notes: *Primary-only includes only incident cases (excluding patients with prior outcome history); Both events includes all patients regardless of prior outcome history. RD calculated as semaglutide risk minus tirzepatide risk in percentage points. Delta RD = RD_Both - RD_Primary. Attenuation ratio = RD_Primary/RD_Both when RD_Both >0. Values represent methodological sensitivity assessment of outcome definition impact.* **Abbreviations:** *RD, risk difference; TIA, transient ischemic attack.*

Supplementary Table 5: Complete Effect Estimates with Precision Metrics.

Outcome	Time	Diabetes Status	Risk Ratio (95% CI)	Odds Ratio (95% CI)	Risk Difference, % (95% CI)
**Atrial Fibrillation**	One-Year	Diabetic	0.881 (0.832, 0.934)	0.875 (0.823, 0.929)	0.72 (0.40, 1.10)
		Non-diabetic	0.762 (0.704, 0.824)	~0.757 (0.699, 0.818)	0.69 (0.60, 0.90)
	Three-Year	Diabetic	0.773 (0.737, 0.811)	0.747 (0.710, 0.786)	1.79 (1.50, 2.10)
		Non-diabetic	0.743 (0.690, 0.801)	0.724 (0.672, 0.780)	0.74 (0.60, 0.90)
**Heart Failure**	One-Year	Diabetic	0.822 (0.783, 0.864)	0.810 (0.767, 0.854)	1.47 (1.10, 1.80)
		Non-diabetic	0.595 (0.546, 0.648)	~0.586 (0.537, 0.638)	0.96 (0.80, 1.10)
	Three-Year	Diabetic	0.740 (0.710, 0.772)	0.686 (0.657, 0.718)	3.05 (2.70, 3.40)
		Non-diabetic	0.583 (0.538, 0.632)	0.564 (0.521, 0.611)	1.21 (1.00, 1.40)
**Acute Myocardial Infarction**	One-Year	Diabetic	0.766 (0.678, 0.867)	0.764 (0.674, 0.865)	0.35 (0.20, 0.50)
		Non-diabetic	0.579 (0.474, 0.707)	0.578 (0.473, 0.706)	0.19 (0.10, 0.20)
	Three-Year	Diabetic	0.550 (0.502, 0.603)	0.506 (0.462, 0.555)	1.37 (1.20, 1.50)
		Non-diabetic	0.484 (0.400, 0.587)	0.488 (0.404, 0.589)	0.28 (0.20, 0.40)
**Ischemic Stroke/TIA**	One-Year	Diabetic	0.686 (0.586, 0.804)	0.684 (0.583, 0.803)	0.30 (0.20, 0.40)
		Non-diabetic	0.560 (0.450, 0.695)	0.559 (0.449, 0.694)	0.19 (0.10, 0.30)
	Three-Year	Diabetic	0.579 (0.514, 0.651)	0.556 (0.494, 0.626)	0.70 (0.60, 0.80)
		Non-diabetic	0.523 (0.426, 0.642)	0.463 (0.379, 0.565)	0.27 (0.20, 0.30)
**Hemorrhagic Stroke**	One-Year	Diabetic	0.644 (0.446, 0.929)	0.644 (0.446, 0.929)	0.07 (0.00, 0.10)
		Non-diabetic	0.574 (0.351, 0.940)	0.574 (0.351, 0.940)	0.03 (0.00, 0.10)
	Three-Year	Diabetic	0.565 (0.437, 0.730)	0.565 (0.437, 0.731)	0.14 (0.10, 0.20)
		Non-diabetic	0.492 (0.315, 0.766)	0.492 (0.315, 0.766)	0.04 (0.00, 0.10)

Notes: *All estimates from propensity score-matched cohorts. Risk ratio and odds ratio <1.0 indicate lower risk with tirzepatide compared with semaglutide. Risk difference calculated as semaglutide risk minus tirzepatide risk in percentage points; positive values favor tirzepatide. Complete precision metrics provided for methodological transparency.* ***Abbreviations:*** *CI, confidence interval; TIA, transient ischemic attack.*
